# Modelling non-alcoholic fatty liver disease in human hepatocyte-like cells

**DOI:** 10.1098/rstb.2017.0362

**Published:** 2018-05-21

**Authors:** Marcus J. Lyall, Jessy Cartier, John P. Thomson, Kate Cameron, Jose Meseguer-Ripolles, Eoghan O'Duibhir, Dagmara Szkolnicka, Baltasar Lucendo Villarin, Yu Wang, Giovanny Rodriguez Blanco, Warwick B. Dunn, Richard R. Meehan, David C. Hay, Amanda J. Drake

**Affiliations:** 1 University/British Heart Foundation Centre for Cardiovascular Science, University of Edinburgh, The Queen's Medical Research Institute, 47 Little France Crescent, Edinburgh EH16 4TJ, UK; 2 MRC Human Genetics Unit, IGMM, Western General Hospital, Crewe Road, Edinburgh EH4 2XU, UK; 3 MRC Centre for Regenerative Medicine, University of Edinburgh, Edinburgh EH16 4UU, UK; 4 Phenome Centre Birmingham, School of Biosciences and Institute of Metabolism and Systems Research, University of Birmingham, Edgbaston, Birmingham B15 2TT, UK

**Keywords:** NAFLD, steatosis, stem cells, hepatocytes, 5-hydroxymethylcytosine, mitochondrial dysfunction

## Abstract

Non-alcoholic fatty liver disease (NAFLD) is the most common cause of liver disease in developed countries. An *in vitro* NAFLD model would permit mechanistic studies and enable high-throughput therapeutic screening. While hepatic cancer-derived cell lines are a convenient, renewable resource, their genomic, epigenomic and functional alterations mean their utility in NAFLD modelling is unclear. Additionally, the epigenetic mark 5-hydroxymethylcytosine (5hmC), a cell lineage identifier, is rapidly lost during cell culture, alongside expression of the Ten-eleven-translocation (*TET*) methylcytosine dioxygenase enzymes, restricting meaningful epigenetic analysis. Hepatocyte-like cells (HLCs) derived from human embryonic stem cells can provide a non-neoplastic, renewable model for liver research. Here, we have developed a model of NAFLD using HLCs exposed to lactate, pyruvate and octanoic acid (LPO) that bear all the hallmarks, including 5hmC profiles, of liver functionality. We exposed HLCs to LPO for 48 h to induce lipid accumulation. We characterized the transcriptome using RNA-seq, the metabolome using ultra-performance liquid chromatography-mass spectrometry and the epigenome using 5-hydroxymethylation DNA immunoprecipitation (hmeDIP) sequencing. LPO exposure induced an NAFLD phenotype in HLCs with transcriptional and metabolomic dysregulation consistent with those present in human NAFLD. HLCs maintain expression of the *TET* enzymes and have a liver-like epigenome. LPO exposure-induced 5hmC enrichment at lipid synthesis and transport genes. HLCs treated with LPO recapitulate the transcriptional and metabolic dysregulation seen in NAFLD and additionally retain *TET* expression and 5hmC. This *in vitro* model of NAFLD will be useful for future mechanistic and therapeutic studies.

This article is part of the theme issue ‘Designer human tissue: coming to a lab near you’.

## 1. Introduction

Non-alcoholic fatty liver disease (NAFLD) now affects around 25–33% of the population and up to 75% of obese individuals in developed countries [1,2]. NAFLD encompasses a spectrum of liver disease and while simple steatosis is considered relatively benign, it can progress to non-alcoholic steatohepatitis (NASH), fibrosis and cirrhosis, and approximately 25% of those with cirrhosis will develop hepatocellular carcinoma (HCC) [3,4]. Progression is highly variable between individuals, with only a minority of individuals reaching end-stage liver disease and/or developing HCC [5]. Such inter-individual variability, coupled with a lack of understanding of the underlying mechanisms, has limited the development of effective therapeutic interventions.

Studies in whole liver may be confounded by shifting cell populations, and therefore the possibility of recapitulation of multiple facets of human NAFLD in a single cell-type *in vitro* would be a key advance, permitting the dissection of disease processes in mechanistic studies and enabling high-throughput screening for new medicines. Exposure of cancer cell lines to saturated (typically palmitate) or unsaturated (typically oleate) long chain fatty acids results in steatosis accompanied by upregulation of cytokines, interruption of insulin signalling, increased reactive oxygen stress and apoptosis signalling [6–9]. However, while hepatic cancer-derived cell lines are a convenient, renewable resource, they have many genomic and functional alterations so that their utility in modelling diseases of overnutrition is unclear [10,11]. Primary hepatocytes isolated from human tissue have also been employed to model disease and predict drug toxicity, however, purification is technically challenging and isolated cells rapidly lose phenotype in cell culture [12]. Furthermore, the gene expression changes that occur with adaptation to culture resemble alterations present in liver disease and may additionally be influenced by the donor's hepatic phenotype and pharmacological history [13]. The renewable, pluripotent nature of embryonic stem cells (ESCs) presents an opportunity for human differentiated tissue to be reproducibly generated *in vitro* for the investigation of human disease processes. Exposure of ESCs to a refined regime of chemical and biological growth stimuli over a three to four week period allows robust, scalable generation of hepatocyte-like cells (HLCs) that exhibit a similar morphology, function and transcriptome to human hepatocytes [13,14]. As a consequence, HLCs have been used extensively in mechanistic studies of drug-induced liver injury, hepatitis viral replication, fetal xenobiotic exposure and the characterization of inherited disorders of lipid metabolism [15–18].

Recent studies in human liver biopsy specimens and in animal models suggest that epigenetic dysregulation could play a role in NAFLD pathogenesis and progression [19–21]. Our studies suggest that the cytosine modification 5-hydroxymethylcytosine (5hmC) may be useful as a biomarker of both normal and abnormal liver physiology [22,23]. However, 5hmC is rapidly lost during somatic cell adaptation to culture and in all tested hepatocyte cell lines [24,25], making it difficult to undertake meaningful epigenetic analyses (particularly 5hmC) in cultured cell line models. Here, we show that HLCs derived from human ESCs retain the ability to form 5hmC, providing a non-neoplastic and renewable source of human cells for liver research. Additionally, exposure of HLCs to a nutrient cocktail of lactate, pyruvate and octanoic acid produces a robust NAFLD-like phenotype *in vitro*. We believe this model will be of importance for further mechanistic studies of NAFLD on defined genetic backgrounds.

## 2. Material and methods

### (a) Embryonic stem cell derived hepatocyte differentiation and LPO treatment

**Figure 1.**
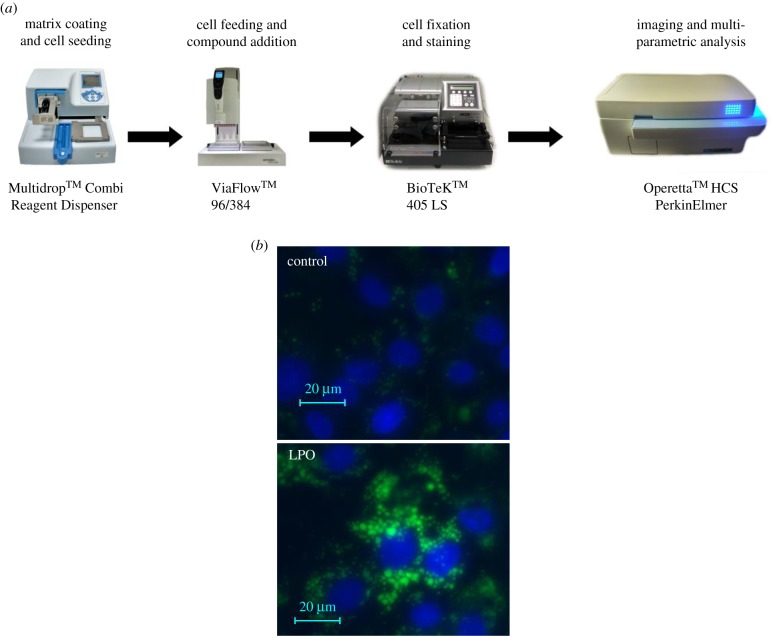

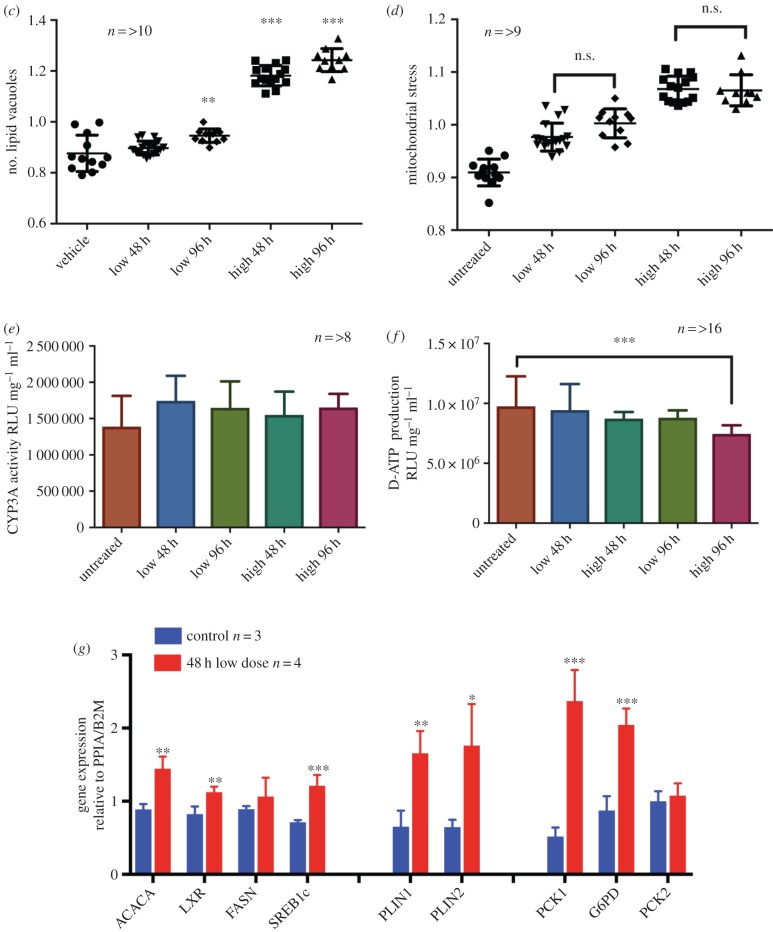
Incubation of HLCs with LPO at low or high dose for 48 or 96 h induces steatosis, respectively. (*a*) Experimental pathway for automated high-throughput cell differentiation, cell staining and image analysis. (*b*) Fluorescence microscopy demonstrating BoDIPY staining of neutral lipid vacuoles within HLC following exposure to LPO. (*c*) LPO induces a dose- and time-dependent increase in lipid vacuoles in HLCs. (*d*) Exposure to LPO increases mitochondrial stress in a dose-dependent manner at 48 and 96 h. (*e*) LPO treatment does not affect cell functionality as determined by a CYP3A activity luciferase assay. CYP3A activity data are reported as relative light units (RLU) normalized to protein content. (*f*) 96 h high-dose LPO increases apoptosis as determined by ATP production assay. Data are reported as RLU normalized to protein content. (*g*) qPCR of mRNA levels of genes relevant to human NAFLD in control (Con, blue) versus 48 h LPO-treated (red) HLCs. Data are expressed relative to the mean of control genes PPIA/B2M and were analysed by one-way ANOVA with Bonferroni correction. The minimum number of biological replicates is shown for each experiment. *p < 0.05, **p < 0.01, ***p < 0.001, n.s. = non-significant.

Female H9 ESCs were differentiated into HLCs as previously described [14]. At day 20 of differentiation, cells were exposed to sodium l-lactate (L), sodium pyruvate (P) and octanoic acid (O) (all Sigma, Gillingham, UK) at low (L : P : O 10 mM : 1 mM : 2 mM) or high dose (L : P : O: 20 mM : 2 mM : 4 mM) for 48 or 96 h to determine optimal conditions for further study. The automated high-throughput system for HLC differentiation, staining and objective image analysis is depicted in figure 1*a*; for detailed methods, see the electronic supplementary material.

### (b) Transcriptome profiling

RNA was extracted and DNase treated using Qiazol, DNaseI and an RNeasy Minikit (Qiagen, Manchester, UK). RNA labelling was performed on 500 ng RNA using the Illumina Total Prep RNA Amplification Kit (Life Technologies. Paisley, UK). Hybridization of biotinylated RNA to Illumina HT-12 beadchip arrays (control *n* = 4, LPO treated *n* = 6) with Illumina Whole Genome Gene Expression Direct Hybridization Assay (WGGX kit, Illumina, Cambridge, UK) was performed at the University of Edinburgh Genomics Core (Western General Hospital, Edinburgh, UK). Imaging was performed and analysed using the Illumina HiScan platform and genotypes called automatically using GenomeStudio Analysis software version 2011.1. Background subtraction, quartile normalization and differential expression were performed using R version 3.2 with Lumi and Limma package, respectively. Gene ontology was performed using GOstats and KEGG pathway analysis using Pathview (www.Bioconductor.org). Unsupervised clustering was performed using Euclidean distance. Where multiple probes mapped to the same gene, the median was used. Data have been uploaded to EBI-Array Express, accession number E-MTAB-6227.

For validation, 800 ng mRNA were reverse transcribed using the High Capacity cDNA Reverse Transcriptase Kit (Life Technologies, Paisley, UK). Quantitative real-time PCR validation was performed using the Roche Universal Probe Library (Roche, Burgess Hill, UK) or Taqman PCR assay (Life Technologies, Paisley, UK) on the Roche Lightcycler 480 (Roche, Burgess Hill, UK) and normalized to endogenous controls as indicated. Primers are in the electronic supplementary material, table S1*a*.

### (c) Metabolome profiling

Metabolome profiling on cell media and HLCs was undertaken using ultra-performance liquid chromatography-mass spectrometry (UPLC-MS) using a Thermo Scientific Ultimate 3000 UPLC system coupled to an electrospray Q Exactive Focus mass spectrometer. A hydrophilic interaction liquid chromatography (HILIC) assay to investigate water-soluble metabolites and a C_18_ reversed phase method to investigate lipid metabolites were applied. Univariate and multivariate data analyses were performed in MetaboAnalyst 3.0 [26], including principal components analysis (PCA), Mann Whitney *U*-tests or Kruskal–Wallis tests to identify metabolites demonstrating a statistically significant change in relative concentrations between two or three biological classes. Fold changes were calculated by division of the mean peak response for one biological class by the mean peak response of the second biological class. For detailed methods, see the electronic supplementary material.

### (d) 5hmC profiling

For slot blotting, serial dilutions of extracted DNA were blotted onto Hybond N+ nitrocellulose membrane (Amersham, Buckinghamshire, UK) using a slot blot manifold (HSI, GE Healthcare, Buckinghamshire UK), washed in 2xSSC buffer and dried overnight. Membrane was then probed with 1 : 10 000 anti 5hmC antibody (Active Motif, cat:39769) for 1 h at room temperature followed by goat anti-rabbit IRDye 800CW (1 : 10 000) for 1 h at room temperature and subsequent imaging on the Li-cor Odyssey infrared imaging system (both Li-cor, Cambridge, UK). DNA loading was confirmed by staining with 0.02% methylene blue in 0.3 M sodium acetate (pH 5.2) and destaining with ddH_2_O.

5hmC DNA immunoprecipitation (hmeDIP) was performed and libraries sequenced on an Ion Torrent semiconductor sequencer using the Ion PI™ Hi-Q™ Sequencing Kit and an Ion PI™ Chip Kit v3 (Thermo Fisher Scientific, Paisley, UK) to a depth of approximately 30 million reads (see the electronic supplementary material). Raw sequencing data were quality controlled, filtered and aligned using Ion Torrent suite software (Life Technologies, Paisley, UK) and then normalized to total reads in R using bespoke scripts. Relative 5hmC levels per 150 bp window were determined using the ‘sliding windows’ function on the Galaxy server. Genomic annotation data for human (hg19) analyses were downloaded from the University of California Santa Cruz Genome Bioinformatics Resource. Further details on hmeDIP bioinformatic processing can be found in Thomson *et al.* [27]. Raw and processed data files are available for download from the GEO repository accession number GSE109139.

### (e) Statistics

Prism GraphPad software (GraphPad Software Inc.) was used for analysis of qPCR and cell culture variables. Data were routinely analysed for outliers and normality of distribution. Non-parametric data or data with significant outliers were log_10_ transformed. If data remained non-parametric, a non-parametric test was used as indicated. Data point and bar graphs are shown as mean ± s.e.m.

## 3. Results

### (a) Exposure of hepatocyte-like cells to lactate, pyruvate and octanoate leads to steatosis and upregulation of genes implicated in non-alcoholic fatty liver disease pathogenesis

Human embryonic stem cells were differentiated to HLCs using a semi automated system (figure 1*a*). At day 21, HLCs formed a stable sheet of cells in a two-dimensions exhibiting polygonal morphology and cytoplasmic lipid droplets (electronic supplementary material, figure S1*a*). During differentiation, cells displayed a reduction in the pluripotency markers, including: POU class 5 homeobox 1 (POU5F1) and Nanog homeobox (NANOG) with a concurrent increase in the hepatocyte-specific markers ALB and hepatocyte nuclear factor 4 alpha (HNF4*α*) (electronic supplementary material, figure S1*b*). At day 21, CYP1A2 and CYP3A4 activity and secreted human albumin were evident and consistent with previous reports [28,29] (electronic supplementary material, figure S1*c,d*).

HLC LPO exposure induced a dose- and time-dependent increase in lipid vacuole generation (figure 1*b,c*). LPO exposure increased mitochondrial stress in a dose-dependent manner at both 48 and 96 h of exposure as determined by an increase in mitochondrial inner membrane potential allowing uptake of MitoTracker Deep Red dye (https://www.nature.com/protocolexchange/protocols/3673#/equipment figure 1*d*). While LPO treatment did not affect cell functionality as determined by CYP3A assay (figure 1*e*), high-dose LPO over a longer duration increased apoptosis (figure 1*f*). Thus, to avoid studying secondary effects associated with cell death, we opted for the lowest dose of LPO treatment for 48 h in all further analyses. qPCR analysis showed that LPO exposure consistently stimulated the expression of multiple putatively causal genes in NAFLD pathological processes, including: fatty acid synthesis (ACACA, LXR, SREB1c), gluconeogenesis (PCK, G6PD) and lipid vesicular transport proteins Perilipin 1 and 2 (PLIN1, PLIN2) (figure 1*g*).

### (b) 48 h lactate, pyruvate and octanoic acid exposure induces transcriptional dysregulation

**Figure 2.**
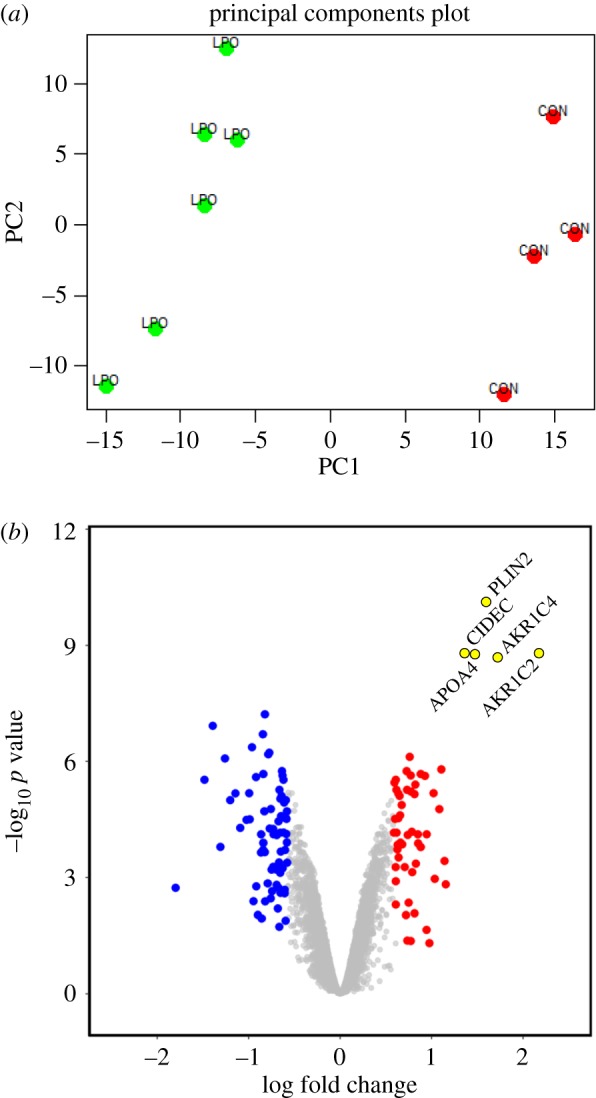
LPO-treated HLCs show transcriptional derangement. (*a*) Principal component (PC) analysis of transcriptome from microarray studies following 48 h low-dose LPO exposure shows clustering of control (CON) and LPO-treated HLCs. (*b*) Transcriptome analysis volcano plot of mRNA expression following LPO treatment. PLIN2, CIDEC, APOA4, AKR1C2 and AKR1C4 are highly induced. Blue/red colours indicate 1.5-fold down- and upregulated genes, adjusted *p*-value < 0.05. *n* = 4 control and *n* = 6 LPO.

We examined changes in transcription following 48 h LPO exposure using the Illumina HT-12 beadchip microarray (Illumina, San Diego, USA). PCA demonstrated clear separation between treated and control populations (figure 2*a*). 48 h LPO treatment resulted in the dysregulation of 2618 transcripts (adjusted *p*-value < 0.05, Benjamini Hochberg false discovery rate (FDR)), with upregulation of 1309 transcripts corresponding to 1083 genes and downregulation of 1309 transcripts mapping to 980 genes (greater than 10% transcriptional change, Benjamini–Hochberg adjusted *p*-value 0.05; figure 2*b* and electronic supplementary material, table S2). The magnitude of change is consistent with a number of human datasets [20,30]. GO terms with the greatest enrichment in upregulated transcripts included ‘GO:0044255’ ‘cellular lipid metabolic process’, ‘GO:0090208’ ‘positive regulation of triglyceride metabolic process’ and ‘GO:1901617’ ‘organic hydroxyl compound biosynthetic process' (electronic supplementary material, figure S2*a*). A specific cluster of five genes were induced to a particularly high degree of significance: the lipid vesicle transport proteins Cell Death-Inducing DFFA-Like Effector C (CIDEC), PLIN2, Apolipoprotein A4 (APOA4) and the steroid hormone synthesis enzymes aldo-keto reductase family 1, members C2, C4 (electronic supplementary material, table S2).

### (c) Lactate, pyruvate and octanoic acid-induced transcriptional and metabolic dysregulation is consistent with mitochondrial dysfunction

We undertook metabolomic profiling in cells and media following 48 h of LPO exposure using UPLC-MS. PCA demonstrated clear separation between treated and control populations (electronic supplementary material, figure S3*a,b*). Mitochondrial dysfunction is a central feature of NAFLD and the degree of dysfunction may be important in determining the risk of progression from steatosis to NASH [31]. Mitochondrial oxidative function encompasses many processes, including tricarboxylic acid (TCA) cycle metabolism, β-oxidation, ketogenesis, respiratory chain activity and ATP synthesis. In agreement with previous studies in mice and human liver tissue [32,33], LPO exposure induced significant TCA cycle dysregulation with altered expression of a number of enzymes, including downregulation of isocitrate dehydrogenase type 1 and 2 (IDH1 and 2) and malate dehydrogenase type 1 (MDH1); and upregulation of IDH3A, alpha-ketoglutarate dehydrogenase (OGDH) and succinate dehydrogenase subunit A (SDHA) (electronic supplementary material, figure S4*a*; table 1). We also observed an increase in the metabolite oxalosuccinate, an intermediate formed during the oxidative carboxylation of isocitrate to alpha-ketoglutarate (electronic supplementary material, figure S4*a*).

**Table 1.** Microarray analysis showing dysregulated transcripts in KEGG pathways relevant to energy metabolism. Adj *p*-value = adjusted *p*-value (Benjamini–Hochberg correction).

KEGG pathway	gene name	log_2_FC	Adj *p*-value
TCA cycle hsa00020	*IDH2*	−0.285	0.004
	*IDH3A*	0.271	0.017
	*MDH1*	−0.192	0.025
	*OGDHL*	0.360	0.001
	*SDHA*	0.236	0.045
	*PCK1*	0.635	0.007
fatty acid degradation hsa00071	*ACSL1*	0.459	0.007
	*CPT1A*	0.281	0.001
	*ACADM*	0.324	0.009
	*ACADVL*	0.727	0.000
	*ACAT1*	0.213	0.008
	*ADH1A*	−0.305	0.008
	*ALDH2*	−0.274	0.026
	*CYP4A11*	0.814	0.008
oxidative phosphorylation hsa00190	*NDUFB3*	−0.213	0.023
	*SDHC*	−0.252	0.031
	*SDHA*	0.236	0.045
	*UQCRC1*	0.226	0.019
	*ATP5J2*	−0.225	0.013
	*ATP5I*	−0.310	0.005
	*ATP6V0E2*	0.308	0.005
	*ATP6V0D1*	−0.206	0.018
	*COX11*	0.173	0.031
glycolysis/gluconeogenesis hsa00010	*GLYCTK*	−0.454	0.004
	*AKR1A1*	−0.170	0.040
	*AKR1B10*	1.139	0.000
	*PLPP2*	0.385	0.006
	*CEL*	−0.319	0.014
NAFLD hsa04932	*LEP*	−0.793	0.001
	*CXCL8*	0.772	0.043
	*CDC42*	−0.500	0.001
	*IRS2*	0.485	0.001
	*CDC42*	−0.463	0.000
	*JUN*	0.427	0.009
	*PIK3R1*	−0.349	0.003
	*ADIPOR2*	0.292	0.001
	*AKT1*	−0.193	0.017
	*ERN1*	0.180	0.038

**Figure 3.**
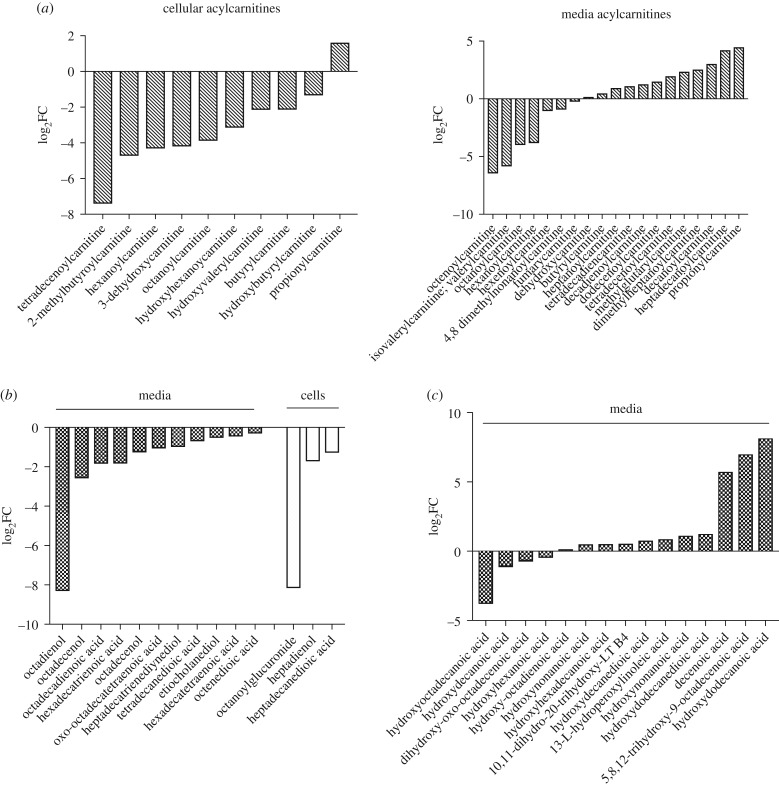
LPO treatment of HLCs induces dysfunction of oxidative phosphorylation. (*a*) Metabolomics analysis demonstrating that LPO induces a reduction in intracellular acylcarnitines and dysregulation of acyl carnitine species (log_2_FC versus control, FDR < 0.01). (*b*) LPO treatment induced a reduction in native fatty acid species in media (hatched bars) and cell (open bars) and (*c*) an accumulation of *ω* oxidation products.

Breakdown of free fatty acids in liver involves β-oxidation. Initial transport of unmodified long-chain fatty acids through the mitochondrial membrane requires the addition of carnitine by carnitine palmitoyl transferase 1 (CPT1), generating acyl carnitine species that are shuttled into mitochondria by carnitine–acylcarnitine translocase. Consistent with previous data [31], LPO-induced steatosis was associated with activation of the β-oxidation transcriptional pathway (electronic supplementary material, figure S4*b*), with upregulation of long-chain-fatty acid-CoA ligase 1 (ACSL1), CPT1A, acyl coenzyme A dehydrogenase (ACADM), very long-chain-specific acyl-CoA dehydrogenase (ACADVL), and acetyl CoA acetyltransferase (ACAT1) (electronic supplementary material, figure S4*b*). Defective β-oxidation in NAFLD results in the accumulation of acyl carnitines and diacylglycerols [31], and in support of this we observed substantial dysregulation of carnitine and acyl carnitine metabolites and glycerol species in cells and media with LPO (figure 3*a*). Further, we observed marked alterations in fatty acids, oxidized fatty acids and acyl glycine species in LPO-treated cells and media (figure 3*b*). Defective oxidative phosphorylation is also feature of NAFLD [31] and we identified altered expression of transcripts involved in all five of the mitochondrial complexes in LPO-treated HLCs (table 1).

When β-oxidation is saturated/dysfunctional, surplus fatty acids can be degraded through microsomal ω-oxidation, to produce hydroxylated fatty acids. Crucially, this comparatively inefficient process increases cellular oxidative stress, a key pathological mechanism in NAFLD progression. LPO treatment was associated with activation of ω-oxidation, with upregulation of cytochrome P450 4A11 (CYP4a11) (table 1) and accumulation of hydroxylated fatty acids in cells and media (figure 3*c*). An alternative method of fatty acid clearance involves the synthesis and sequestration of triglycerides within lipid vesicles, the primary histological finding in hepatic steatosis. Our findings of an LPO-induced increase in glycerol-3-phosphate within cell media with induction of phospholipid phosphatase 2 (PLPP2) and suppression of the lipolytic enzyme carboxyl ester lipase (CEL) support an increase in triglyceride synthesis in LPO-treated HLCs (table 1). In addition to this, upregulation of the lipid vesicle transport proteins PLIN1, PLIN2 (figure 1*g*) and CIDEC and the structural lipoprotein APOA4 (figure 2*b*) are reflected in the increase in lipid vacuoles with LPO exposure.

### (d) Other aspects of lactate, pyruvate and octanoic acid-induced transcriptional and metabolic dysregulation are consistent with non-alcoholic fatty liver disease

LPO treatment was associated with increased expression of cytosolic phosphoenolpyruvate carboxykinase (PCK1; table 1; electronic supplementary material, table S2), the rate-limiting enzyme in gluconeogenesis and an important regulator of TCA cycle activity. An increase in gluconeogenesis in LPO-exposed HLCs was supported by increased phosphoenolpyruvate and glucose in cell media (table 2). PCK1 may be an important therapeutic target because knocking down PCK1 can prevent oxidative stress and inflammation in mice on a high-fat diet [33]. Examination of the KEGG pathway for NAFLD (hsa04932) demonstrated transcriptional dysregulation of mediators of insulin resistance (IRS2, P1K3R1, AKT1), steatohepatitis ( JUN, ERN1) and neutrophil infiltration and inflammation (CXCL8) (table 1).

**Table 2.** Metabolite analysis: metabolites from media and cells showing differences between control and LPO-treated HLCs. FDR = false discovery rate correction for multiple testing.

metabolites	sample	KEGG pathway/compound group	fold change	FDR
1H-indole-3-carboxaldehyde	cells	aromatic metabolites	0.264	0.001
*N*'-formylkynurenine			0.116	0.000
butyrylcarnitine		carnitine and acyl carnitine metabolism	0.221	0.001
octanoylcarnitine			0.066	0.000
propionylcarnitine			3.145	0.050
octadecenol		fatty acids and oxidized fatty acids	0.161	0.045
arginine	media	arginine metabolism	1.221	0.007
argininosuccinic acid			1.523	0.000
1H-indole-3-acetamide		aromatic metabolite metabolism	1.096	0.008
1H-indole-3-carboxaldehyde			1.252	0.001
lactic acid			0.517	0.000
5-hydroxy-l-tryptophan			0.684	0.000
5-hydroxy-*N*-formylkynurenine			0.777	0.001
5-hydroxyindoleacetic acid			1.888	0.000
dihydroxyindole			0.833	0.004
formyl-5-hydroxykynurenamine			1.185	0.006
hydroxyphenylacetylglycine			1.803	0.000
indole			0.670	0.000
*N*'-formylkynurenine			36.59	0.000
phenylethylamine			0.006	0.000
tryptophan			1.683	0.001
butyrylcarnitine		carnitine and acyl carnitine metabolism	1.450	0.000
carnitine			1.139	0.001
hydroxy-hexadecenoylcarnitine			3.116	0.005
hydroxy-tetradecadiencarnitine			2.684	0.000
hydroxy-tetradecenoylcarnitine			5.062	0.001
octanoylcarnitine			0.060	0.000
propionylcarnitine			23.13	0.000
thiocysteine		cysteine and methionine metabolism	5.485	0.001
dihydroxy-oxo-octadecenoic acid		fatty acid and oxidized fatty acids	0.566	0.001
hydroxy-octadienoic acid			1.148	0.005
hydroxydecanoic acid			0.428	0.001
hydroxydodecanoic acid			295.9	0.003
hydroxyhexadecanoic acid	media		1.498	0.006
hydroxynonanoic acid			1.481	0.000
octadecenol			0.403	0.006
octenedioic acid			0.780	0.001
glucose		glycolysis/gluconeogenesis hsa00010	1.179	0.000
glycerol-3-phosphate			37.34	0.001
glyceric acid		glycolysis	0.644	0.003
lactic acid			0.577	0.000
phosphoenolpyruvic acid			1.474	0.004
phosphoglyceric acid			1.416	0.000
imidazolepropionic acid			histidine metabolism	86.98	0.000
imidazole-4-acetaldehyde		0.004		0.000
7-methylguanosine		purine and pyrimidine metabolism	56.27	0.000
cytidine			0.587	0.004
deoxycytidine			0.438	0.004
dihydroxypurine			0.606	0.000
glutamine		TCA cycle and oxidative phosphorylation	1.298	0.003
oxalosuccinic acid			1.325	0.002
2-methyl-1-hydroxypropyl-ThPP		valine, leucine and isoleucine metabolism	1.162	0.001
isopropylmaleate			2.590	0.005
leucine			594.2	0.000
S-(2-methylpropionyl)-dihydrolipoamide-E			1.188	0.001

### (e) Hepatocyte-like cells maintain 5hmC and *TET* enzyme expression in culture

**Figure 4.**
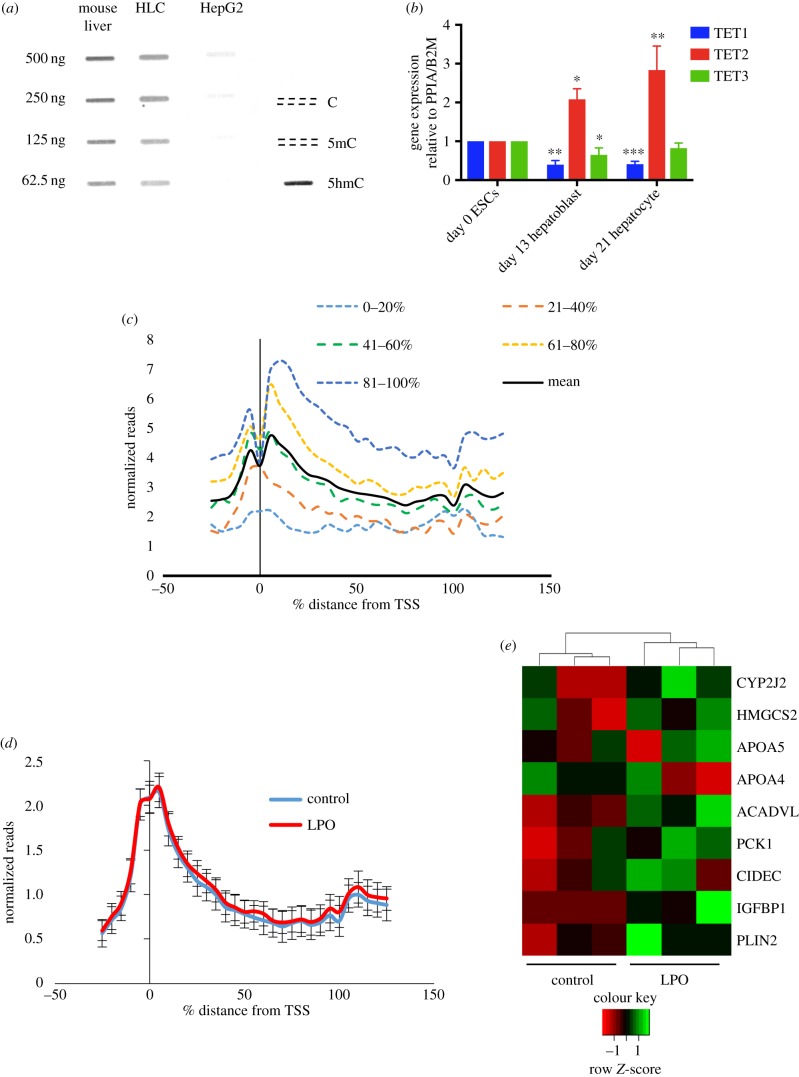
HLCs retain *TET* expression and 5hmC and demonstrate genic 5hmC enrichment in activated lipid synthesis and transport genes on LPO exposure. (*a*) 5hmC immune slot/blot of mouse liver, HLCs and HepG2 cells. Oligonucleotides of the APC gene promoter were used as controls. (*b*) qPCR of TET isoform mRNA expression during differentiation. Values are normalized to internal controls PPIA and B2M and expressed as fold change from undifferentiated ESCs. TET1 and TET3 expression decreases and TET2 expression increases during hepatic differentiation (*p < 0.05, **p < 0.01, ***p < 0.001 one-way ANOVA with Bonferroni multiple test correction versus ESCs). (*c*) Sliding window analysis of hmeDIP-seq displaying 5hmC profiles of HLCs stratified by expression quintile in relation to relative gene length (mean of two separate hmeDIP-seq experiments). (*d*) Sliding window analysis of all genes in control and LPO-treated HLCs (*n* = 3/group) shows no differences in global 5hmC levels. Error bars = s.d. (*e*) Heatmap analysis and unsupervised clustering of change in genic 5hmC specifically over induced genes of lipid synthesis and transport following LPO exposure.

Global epigenetic DNA modification levels and patterns are frequently altered in cultured cells, particularly 5hmC, which is vastly reduced genome-wide [24]. To test if our system maintained a normal 5hmC landscape, we carried out an antibody-based quantitative analysis of 5hmC in HLCs and compared these to ESCs and HepG2 cells. HLCs retain 5hmC levels at equivalent levels to mouse liver, which is much higher than the minimal level observed in HepG2 cells (figure 4*a*). Additionally, although mammalian cell culture is associated with loss of Ten-eleven-translocation (*TET*) enzyme expression [24], the expression of all three *TET* isoforms is maintained in HLCs (figure 4*b*). *TET1* is highly expressed in ESCs and plays a critical role in the maintenance of pluripotency [34] and consistent with this, TET1 mRNA expression is downregulated at the later stages of the hepatic differentiation protocol. *TET2* is the dominant *TET* isoform expressed in liver and expression was appropriately increased during differentiation [35]. In order to validate that HLCs have a liver-like hydroxymethylome and have transitioned from an ESC state, we performed hmeDIP-sequencing (hmeDIP-seq) on HLCs and compared profiles with published datasets for 5hmC in human liver and human ESCs [36,37]. We additionally performed hmeDIP-seq on human kidney to assess tissue-specificity. Accordingly, we observed high levels of 5hmC across key liver genes, e.g. albumin (Alb) exclusively in HLCs and human liver datasets, while low levels of 5hmC were observed over ESC and kidney 5hmC-enriched loci, e.g. the HoxA cluster (electronic supplementary material, figure S5*a,b*). In both human ESCs and liver, high levels of 5hmC are present at transcription start site (TSS), distal promoter and proximal gene body regions [38,39] and bioinformatic analysis of 5hmC datasets in HLCs showed a similar 5hmC profile (figure 4*c*). As in previous studies, 5hmC patterns followed transcriptional states, with highly transcribed genes containing more gene body 5hmC than lowly transcribed genes [40] (figure 4*c*). Thus, 5hmC profiles are consistent with a liver-like epigenome, supporting the utility of this cell line for the study of human metabolic liver disease. We also profiled 5hmC in LPO-treated and control HLCs using hmeDIP-seq. Sliding window analysis showed no global change in 5hmC in LPO-exposed HLCs (figure 4*d*), although we observed some 5hmC enrichment within bodies of induced genes involved in lipid synthesis and transport (electronic supplementary material, figure S6). Such changes allowed for stratification by treatment (figure 4*e*) and highlight the utility of combined transcriptomic and epigenetic profiling to investigate molecular mechanisms.

## 4. Discussion

NAFLD is strongly associated with obesity, insulin resistance (IR), type 2 diabetes (T2DM) and cardiovascular disease [1] and the increasing prevalence of these disorders is a substantial public health burden. Indeed, NAFLD is an early predictor of, and important determinant for, the development of T2DM and the metabolic syndrome [41]. While the use of rodent models of NAFLD to explore mechanisms and test therapies is common, lipid metabolism in mice and humans differs so that the applicability of mouse models to human disease is unclear. The development of high-throughput *in vitro* human-relevant models of NAFLD would facilitate the development of reliable biomarkers of risk, the rapid screening of novel or existing therapies and the accurate prediction of toxicity in response to pharmacological compounds, enabling the development of safe, efficacious and cost-effective medicines.

Human HLCs possess hepatocyte-like morphology, gene expression and function [14]. They can be derived from renewable cell populations at scale, are robust, and are comparable to cryopreserved human hepatocytes in terms of predicting human compound toxicity and model metabolic differences [29]. Exposure of cells to fatty acids has been employed to model NAFLD in cell culture systems with various reports of effects on steatosis, insulin signalling and activation of apoptosis pathways [6,7,9]. The one previous report of the use of HLCs to model NAFLD used oleic acid-induced steatosis and reported perturbation of multiple metabolic pathways, including activation of the proliferator-activated receptor (PPAR) pathway [42]. However, the aetiology of NAFLD may also include the response to nutritional excess, hyperinsulinaemia, oxidative stress, adipokine fluctuations, cytokine signalling and the influence of gut-derived factors [43]. Therefore, more representative models are required. The high-energy substrates lactate and pyruvate have been used in combination with the medium chain fatty acid octanoate and ammonia (LPON) to mimic energy excess in human hepatoblastoma cell lines [44,45]. This cocktail generates hepatic steatosis, impaired mitochondrial function, increased reactive oxygen species and perturbs energy metabolites and the cellular proteome, without compromising cellular viability [43,44]. Here we show that HLC exposure to LPO induces an increase in cellular steatosis and stimulates the expression of multiple genes associated with NAFLD, demonstrating the importance of HLCs as a cell-based model of human NAFLD. We also identified transcriptional and metabolic perturbations consistent with mitochondrial dysfunction, a key feature of NAFLD, with multiple alterations in TCA cycle metabolism, β-oxidation and oxidative phosphorylation.

Recent studies using both genome-wide and candidate gene analysis in human liver biopsy specimens and in animal models have identified alterations in DNA methylation in NAFLD, suggesting that epigenetic dysregulation may play a role in its pathogenesis and progression [19–21]. DNA methylation (5-methylcytosine, 5mC) is important in the regulation of gene expression and plays a key role in transcriptional silencing [46]. By contrast, the cytosine modification 5hmC is enriched over the bodies of expressed genes as well as at enhancer elements and some promoter regions [47–49]. While 5hmC appears to function as part of an active DNA demethylation pathway, catalysed by the *TET* enzymes [50], it may also act as a functional DNA methylation mark. We have suggested that 5hmC patterns may be useful as an identifier of cell/tissue type and a marker of cell state and may be a useful tool to identify novel therapeutics and assess drug response [22,51]. However, 5hmC profiles in whole liver from animal models or human biopsy specimens may be confounded by changes in cell populations as a consequence of the disease process, because 5hmC patterns are highly cell- and tissue-specific. While the use of single cell-types overcomes this problem, there are particular problems with studying 5hmC *in vitro*: 5hmC is rapidly lost during somatic cell adaptation to culture, including in all tested hepatocyte cell lines [24]. Additionally, established HCC cell lines are unsuitable because resident 5mC/5hmC changes are a feature of neoplasia that would confound analyses in these models following interventions to induce steatosis [25,52]. By contrast, we demonstrate that HLCs maintain *TET* expression, have 5hmC profiles consistent with a liver-like epigenome, show 5hmC enrichment within bodies of induced genes and therefore represent a transformative resource with which to study epigenetic changes in human liver disease.

In conclusion, our study shows that HLCs treated with LPO recapitulate the transcriptional and metabolic dysregulation seen in NAFLD and additionally retain expression of the *TET* enzymes and 5hmC. This *in vitro* model of ‘NAFLD in a dish’ using a single cell-type will be useful for future mechanistic and therapeutic studies.

## Supplementary Material

Supplementary materials

## Data accessibility

Transcriptomic data have been uploaded to EBI-Array Express accession number E-MTAB-6227. Raw and processed 5hmC datasets are available from the GEO repository accession number GSE109139.
