# Supplementary material for: Modelling non-alcoholic fatty liver disease in human hepatocyte-like cells
Source: Philos Trans R Soc Lond B Biol Sci. 2018 May 21;373(1750):20170362. doi: 10.1098/rstb.2017.0362 (PMC5974453; doi:10.1098/rstb.2017.0362)
Supplement: Supplementary materials [file rstb20170362supp1.docx]

**Lyall et al Supplementary Material**

**Philosophical Transactions B**

**Modelling non-alcoholic fatty liver disease in human hepatocyte-like cells**

Marcus J Lyall, Jessy Cartier, John P Thomson, Kate Cameron, Jose Meseguer-Ripolles, Dagmara Szkolnicka, Baltasar Lucendo Villarin, Yu Wang, Giovanny Rodriguez Blanco, Warwick B Dunn, Richard R Meehan, David C Hay and Amanda J Drake

**Contents:**

1. **Supplementary methods**
2. **Supplementary figures**
3. **Supplementary tables**
4. **Supplementary references**

**1. Supplementary Methods**

*Cell culture, quantification of lipid vacuoles and analysis of mitochondrial stress in HLCs*

Gene expression, lipid staining, epigenetic and metabolomics experiments were conducted utilising matrigel (Corning, Tewksbury, USA).  Experiments performed which examine lipid vacuole formation, mitochondrial stress and ATP production were performed on laminin 521 (BioLamina, Sundbyberg, Sweden).

For quantification of lipid vacuoles and analysis of mitochondrial stress, HLCs were cultured in a 96 well format. After the LPO induction, cells were stained with the cell painter assay adapted from *Bray et al* (1). Briefly, cells were stained with 30μl of MitoTracker Deep Red (500nM stock solution) (ThermoFisher, Cat: M22426) as previously described (1). Cells were then washed with 100μl/well of 1xHBSS (10×; Invitrogen, cat. no. 14065-056) and fixed with 50μl of 4% (wt/vol) Paraformaldehyde (PFA), methanol free (Electron Microscopy Sciences, cat. no. 15710-S) for 20 minutes at room temperature in the dark. Cells were permeabilised with 50 μl/well of 0.1% (vol/vol) Triton X-100 (Sigma-Aldrich, cat. no. T8787) for 15 minutes. Cell membranes were stained with 1X HCS Green CellMask™ Plasma Membrane Stains (Invitrogen, cat no. H32714) and F-Actin was stained with Alexa Fluor™ 546 Phalloidin (ThermoFisher, Cat: A22283) by adding 50μl/well for 30 minutes in the dark at room temperature. Cells were washed with 100μl/well of 1xHBSS and incubated with 50μl/well of NucBlue Live ReadyProbes® Reagent (Molecular Probes R37605) two drops/ml in 1xHBSS for 5 minutes in the dark at room temperature. After the incubation a final wash of 100μl/well of 1xHBSS was done. Images were acquired using the Operetta high content analysis system and Columbus analysis software (Perkin Elmer, Buckinghamshire, UK). Seven fields were acquired across each well to obtain an average representation of the well. Phenotypic screening of untreated and LPO treated cells was done by measuring changes in intensity and texture in the different channels which stained for different organelles of the cell. Normalisation over the median value of the plate was done for all the different parameters analysed.

CYP3A4 and CYP1A2 activity were measured on day 21 of maturation in 96 well (Figure 1E) and 24 well formats (Supplementary Figure 1C) using pGlo technology (Promega, Southampton, UK) according to the manufacturer’s instructions. Briefly, P450-Glo™ CYP1A2 5mM Luciferin-ME substrate was diluted 1:25 and CYP3A4 Luciferin-BE 5mM substrate was diluted 1:20 before assays commenced and for each reaction 120 µl of substrate was used. Cells were incubated with luciferase P450 substrate for 5 hours at 37°C before collection of media. 50µl of media was then incubated with 50µl of luciferase detection reagent for 20 minutes and luminescence measured on a Luminometer (Promega, Southampton, UK). Media in a well with no cells was used as a negative control and subtracted from final readings. Activity is expressed as relative light units per millilitre of media per mg of protein (Pierce BCA assay, Fisher Scientific, Loughborough, UK). Albumin production was assessed at day 21 of maturation using a human albumin ELISA kit (Alpha Diagnostic International, San Antonio, US). This is a well-validated assay in which human albumin only is detected against a standard curve of human albumin serial dilutions (2). Fresh media serves as a blank and is subtracted from the final value thus leaving signal from human albumin measured in the final result. Samples were run in triplicate and analysed on a FLUOStar Omega multi-mode microplate reader (BMG Labtech, Ortenberg, Germany). Protein production was expressed as nanograms of protein per millilitre of medium per cm^2^ of coalescent cells.

*Metabolome profiling*

Cell media (metabolic footprint) was sampled by direct collection of 200μl aliquots followed by centrifugation (12,000xg, 15 minutes) and freezing at -80°C. Prior to cellular collection, media was removed and cells were washed with three aliquots of Phosphate-Buffered Saline. Following this, 0.9ml of a 40/40/20% solution of acetonitrile, methanol and water (Sigma-Aldrich) at a temperature of approximately -40°C was added to each well and plates were frozen at -80°C for 15 min to provide quenching of metabolism and cell lysing. Cells were scraped off the wells and the suspension was centrifuged at 12,000xg (4°C) for 10 min to separate the extraction supernatant from cell pellet. The extraction supernatant was dried applying a vacuum centrifugal evaporator (Thermo Scientific Savant SPD111V speedvac concentrator coupled to a Savant RVT5105 vapour trap). 100μl of media samples were dried applying the same process. Pooled QC samples were prepared for cell media analysis (by pooling of 100μl aliquots of all biological samples) and cell extract analysis (by pooling of 200μl aliquots of all cell extract samples).

Samples were analysed applying ultra-performance liquid chromatography-mass spectrometry (UPLC-MS) using a Thermo Scientific Ultimate3000 UPLC system coupled to an electrospray Q Exactive Focus mass spectrometer. Two assays were applied to increase the number of metabolites detected, a HILIC assay to investigate water-soluble metabolites and a C_18_ reversed phase method to investigate lipid metabolites. The HILIC method applied a Thermo Scientific Accucore 150 Amide HILIC column (100 x 2.1mm, 2.6µm) operated at a temperature of 35°C and a flow rate of 500µL.min-1. Solvent A was 10 mM Ammonium Formate in 95% Acetonitrile/5% water + 0.1% formic acid and solvent B was 10mM Ammonium Formate in 50% Acetonitrile/50% water + 0.1% formic acid. The gradient elution was applied at follows; Start at 99% A for 1 minute, followed by decreases to 85% and 50% at 3 minutes and 6 minutes with a curve of 5 and then a decrease to 5% A at 9 minutes and an increase to 99% A at 10.5 minutes with a curve of 5. The total analysis time was 15 minutes and the injection volume was 2µL. Mass spectral data was collected in positive and negative ion modes separately at a mass resolution of 70,000 (FWHM at m/z 200). Data Dependent Analysis data was acquired for three QC samples to aid metabolite identification. QC samples were analysed 10 times at the start of the run and then after every 6th biological sample with two QC samples analysed after all biological samples had been analysed.

The C_18_ reversed phase method applied a Thermo Scientific Hypersil GOLD C18 column (100 x 2.1mm, 1.9µm) operated at a temperature of 55°C and a flow rate of 400 µL.min-1. Solvent A was 10 mM Ammonium Formate in 60% Acetonitrile/40% water + 0.1% formic acid and solvent B was 10mM Ammonium Formate in 90% isopropyl alcohol/10% acetonitrile + 0.1% formic acid. The gradient elution was applied at follows; Start at 80%A for 0.5 minutes, followed by a decrease to 0% A at 8.5 minutes with a curve of 5 and then an increase to 80% A at 11.5 minutes with a curve of 5. The total analysis time was 15 minutes and the injection volume was 2µL. Mass spectral data was collected in positive and negative ion modes separately at a mass resolution of 70,000 (FWHM at m/z 200). QC samples were analysed 10 times at the start of the run and then after every 6th biological sample with two QC samples analysed after all biological samples had been analysed.

Raw data were converted to the mzML format applying ProteoWizard and then deconvoluted applying the software XCMS (3), operated on an office PC in R using previously described parameters (4). Putative metabolite annotations were provided using the software PUTMEDID_LCMS using a RT window of +/- 2 seconds and a mass error of 5ppm (5). MS/MS mass spectral matches of data to mzCloud (mzCloud–Advanced Mass Spectral Database, https://www.mzcloud.org/, last accessed 30/10/2017) was also performed to enhance confidence of specific metabolite annotations. All metabolites were annotated to MSI Level 2 (6). The data were filtered for quality based on the QC sample data with metabolites with a relative standard deviation >20% or detected in less than 60% of the QC samples being removed (7). Univariate and multivariate data analysis were performed in MetaboAnalyst 3.0 (8). This included Principal Components Analysis (PCA), Mann Whitney U tests or Kruskal-Wallis tests to identify metabolites demonstrating a statistically significant change in relative concentrations between two or three biological classes. Fold changes were calculated by division of the mean peak response for one biological class by the mean peak response of the second biological class.

*5hmC profiling*

For 5hmC DNA immunoprecipitation (hmeDIP) from cultured cells: DNA extraction was performed using the Qiagen DNeasy Blood and Tissue Kit (Qiagen, Crawley, UK) and RNase treated with RNase A (Purelink, Ambion, UK). Human kidney DNA was purchased from Amsbio (Amsbio, Abingdon, Oxfordshire). For hmeDIP, 5µg genomic DNA was sonicated using a Bioruptor (Bioruptor, Ougree, Belgium) to fragments between 100 and 600 base pair length with a mean of 250 to 300 base pairs. 2.5μg of sonicated DNA was diluted to 450μl in TE buffer and denatured for 10 minutes at 90°C in a heat block before cooling for 5 minutes at 4°C and diluting to a final volume of 500μl immunoprecipitation buffer (10mM sodium phosphate (pH 7.0), 140mM NaCl, 0.05% Triton X-100). 10% input was removed at this stage. 1μl of anti-5hmC antibody (Active Motif, La Hulpe, Belgium) was then added to the remaining sample and incubated for 3 hours at 4°C on a rotating wheel. 40μl of Dynabeads protein G (Invitrogen, Paisley, UK) were prewashed with BSA 0.1% in PBS and added to the DNA/antibody mixture for 1 hour at 4°C. Beads were then collected by magnetic rack and washed three times with 1ml of cold IP buffer. Beads were then re-suspended in 250μl of digestion buffer (50mM Tris-HCl pH 8.0, 10mM EDTA pH 8.0, 0.5% SDS) and treated with 20μl of proteinase K 20mg/ml (Roche, UK) in a thermoshaker at 1000rpm, 55°C overnight. Beads were removed using a magnetic rack and the enriched fraction and input samples purified using the Qiagen Qiaquik PCR Purification Kit (Qiagen, UK) with elution in 22μl of water. 1:10 dilution was used for qPCR analysis. For semiconductor sequencing, 10μl was amplified for 18 cycles using a SeqPlex DNA Amplification Kit (Sigma, Gillingham, UK).

100ng of DNA was used to generate a DNA library from each sample using the Ion Xpress Plus Fragment Library Kit (Thermo Fisher Scientific, Paisley, UK). During this process, DNA fragments were end repaired and ligated to specific barcode adaptors before being amplified (8 cycles) and twice purified using the Agencourt AMPure XP PCR Clean Up Kit (Beckman Coulter, High Wycombe, UK). Libraries were quality controlled using the Agilent Bioanalyser DNA HS Kit (Agilent, Santa Clara, USA) and pooled in equimolar pairs prior to template preparation using the Ion PI™ Hi-Q™ OT2 200 Kit (Thermo Fisher Scientific, Paisley, UK) and sequencing on the Ion Torrent semiconductor sequencer using the Ion PI™ Hi-Q™ Sequencing Kit (Thermo Fisher Scientific, Paisley, UK) and an Ion PI™ Chip Kit v3 (Thermo Fisher Scientific, Paisley, UK). For consistency, each sample was sequenced on a PI chip with its own input. Each sample was sequenced to a depth of ~30 million reads prior to quality control. Raw sequencing data were quality controlled, filtered and aligned using Ion Torrent suite software (Life Technologies, Paisley, UK) and then normalised to total reads in R using bespoke scripts. Relative 5hmC levels per 150bp window were determined using the ‘sliding windows’ function on the Galaxy server at IGMM, Western General Hospital, UK. Datasets were compared to published 5hmC datasets for human liver (GSM1716958) (9) and ESCs (GSM936817) (10). Visualisation and hierarchical clustering of data by Z-score heatmap analysis was carried out in R using the Gplots package. Genomic annotation data for human (hg19) analyses were downloaded from the University of California Santa Cruz Genome Bioinformatics Resource. Further details on hmeDIP bioinformatic processing can be found in Thomson *et al* (11). Raw and processed data files are available for download from the GEO repository accession number GSE109139.

**2. Supplementary figures**

**
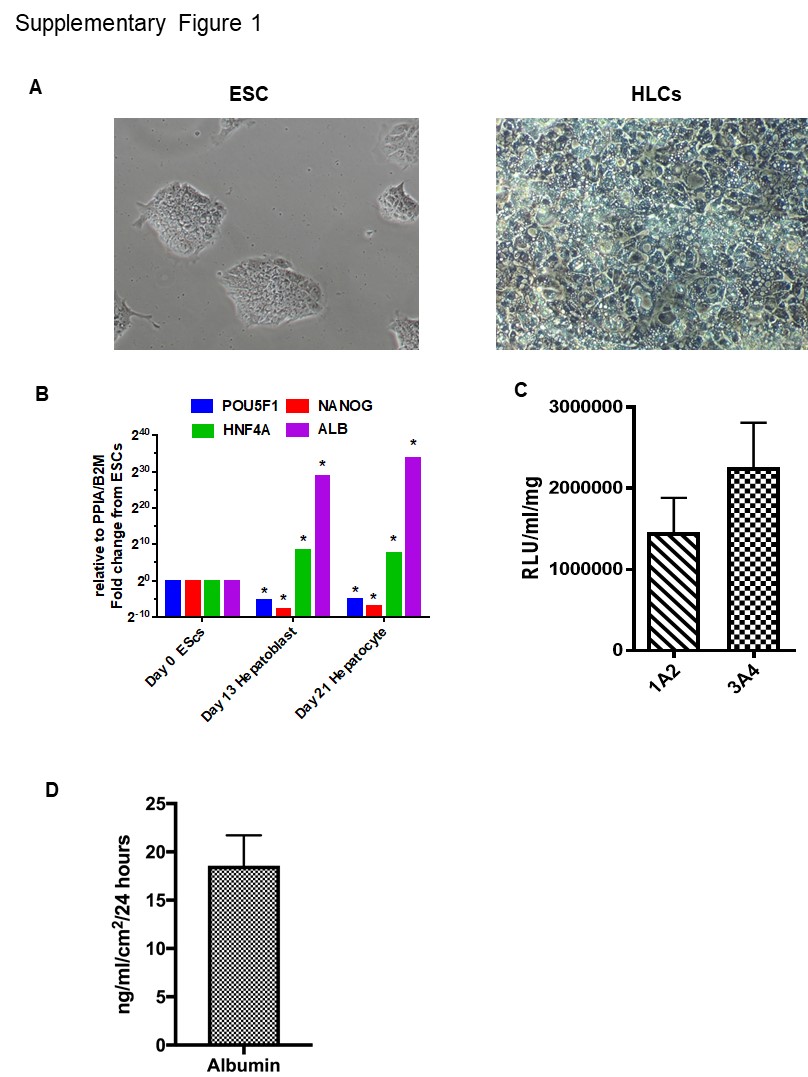
**

*Supplementary Figure 1: ESC differentiation into HLC with maturity markers and functionality.*

(A) Differentiation of H9 ESCs into HLCs showing 10x bright field images of H9 female ESCs (left) and 20 day HLCs (Right). (B) qPCR analysis of mRNA levels of pluripotency and maturation markers of hepatocyte differentiation. Y axis is on a logarithmic scale due to magnitude of differences observed. Values are normalised to internal controls PPIA and B2M and expressed as fold change from undifferentiated ESCs. (*p<0.001 by one-way ANOVA versus ESCs with Bonferroni correction for multiple comparisons) (C) Luciferase assay for CYP 1A2 and CYP 3A4 activity in HLCs reported as relative light units (RLU) normalized to protein content. (D) ELISA of human albumin levels measured on day 21 of differentiation protocol. Albumin levels were calculated from a standard curve of positive human albumin control samples with fluorescence of media control samples subtracted.

*Supplementary Figure 2: Gene ontology analysis*

Gene ontology analysis of upregulated transcripts induced by LPO treatment.


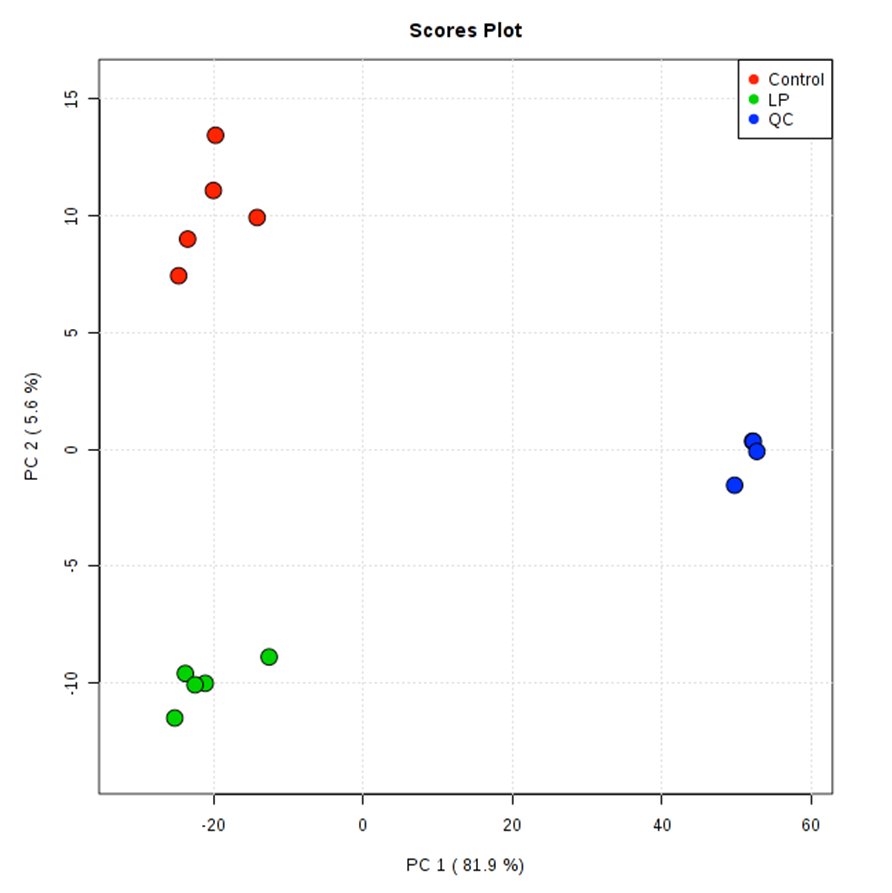


PC 2

B

A

PC 1


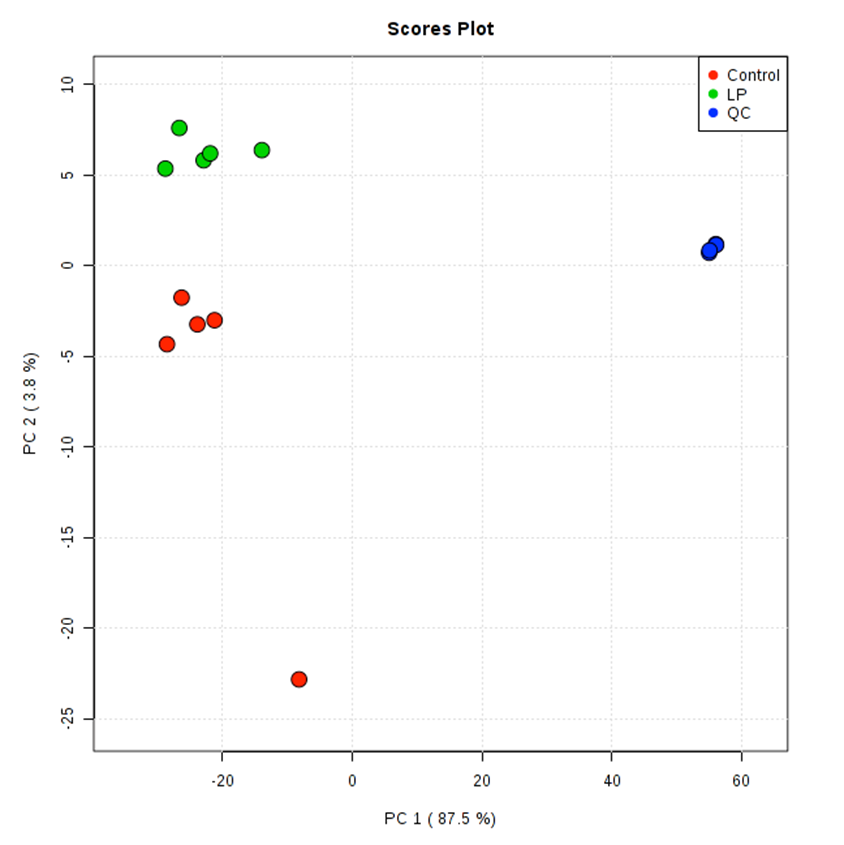


PC 2 (3.8%)

PC 1

*Supplementary Figure 3: LPO exposure results in derangement of the metabolome*

Principle Components (PC) Analysis of metabolome of media from control and LPO treated cells showing clear separation between groups. (A) C18 reversed phase negative ion mode (B) HILIC negative ion mode.

*Supplementary Figure 4: LPO treatment of HLCs induces dysfunction of mitochondrial metabolism and fatty acid degradation*

(A) Integration of metabolomics and microarray analysis. Simplified diagram of TCA cycle and related pathways showing transcriptional and metabolic alterations with LPO exposure. Significantly downregulated genes are shown in yellow and upregulated in orange (adjusted p value< 0.05 Benjamini-Hochberg FDR); LPO-exposure resulted in accumulation of the TCA cycle intermediate oxalosuccinate. (B) Simplified diagram of fatty acid degradation pathways showing activation of multiple steps. Upregulated genes are shown in orange.

**A**

**B**

Control HLC

LPO-treated HLC

Human Liver

Feinberg

GSM1716958)

Human Kidney

Thomson

unpublished)

Human ESC

Park

GSM936817)


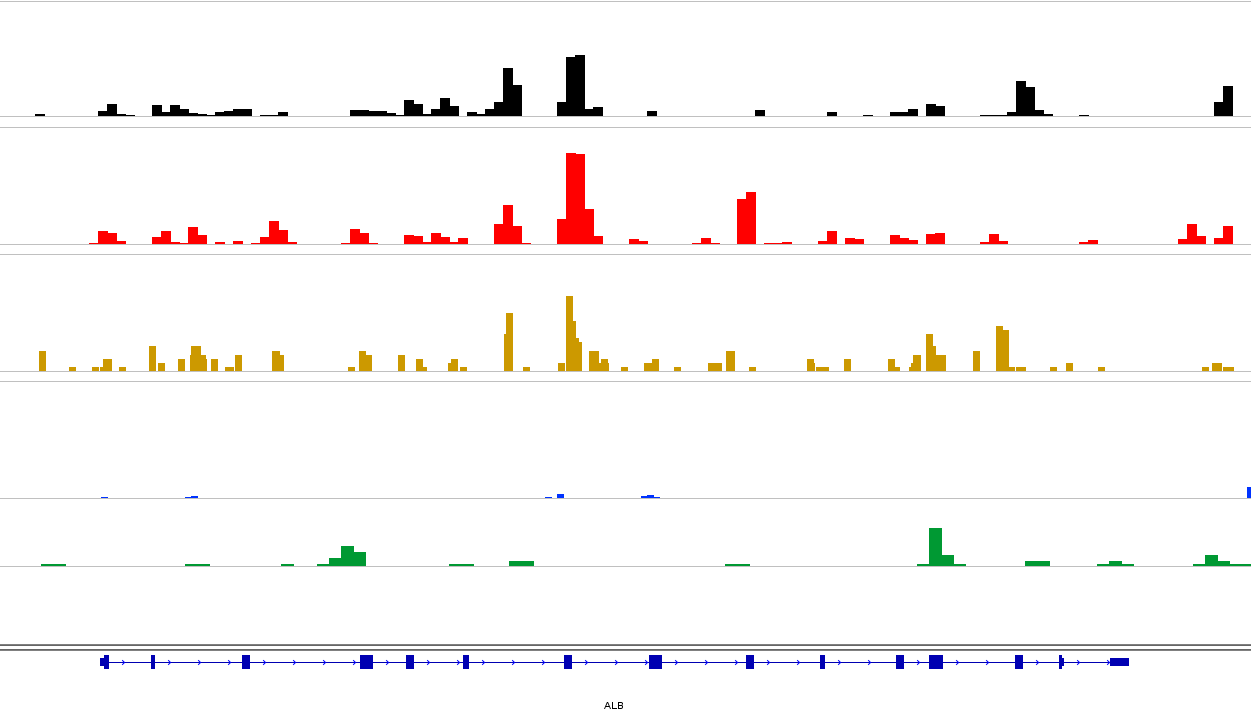

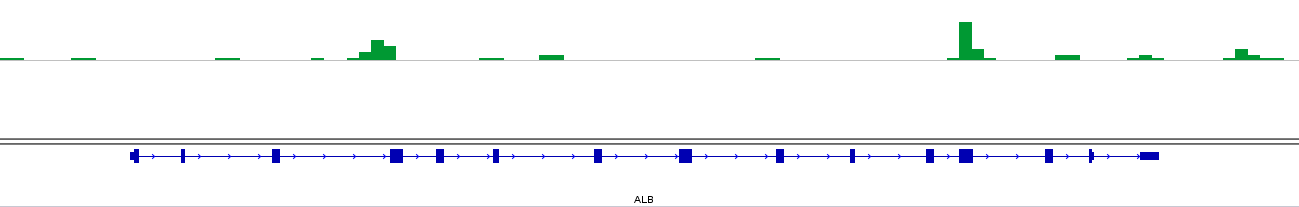


*Alb*

Control HLC

Treated HLC

Human Liver

Feinberg

GSM1716958)

Human Kidney

Thomson

unpublished)

Human ESC

Park

GSM936817)


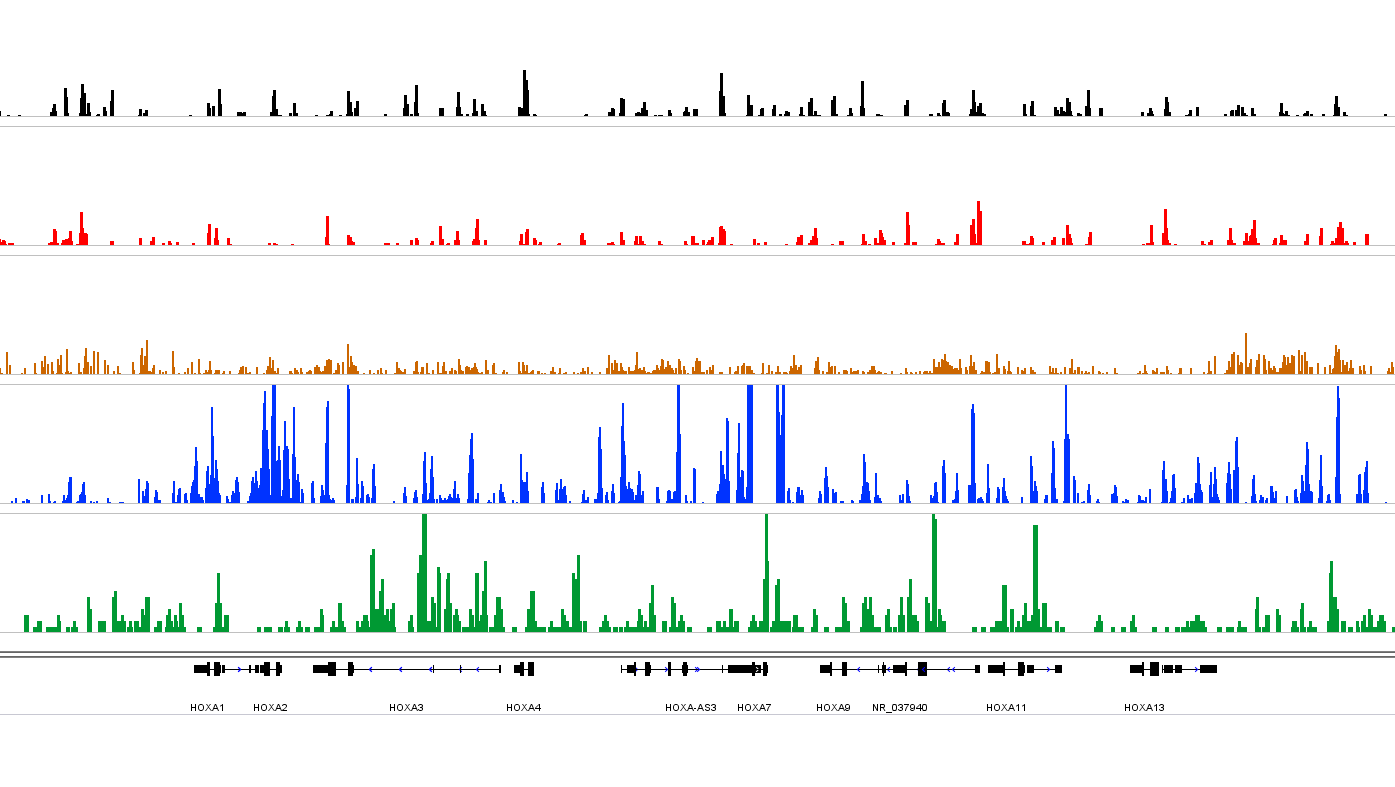


*HoxA cluster*

*Supplementary Figure 5: HLCs show a liver-like hydroxymethylome*

(A) Screenshots of 5hmC levels over 150b windows of the human genome. 5hmC levels over the albumin gene are high in HLCs and human liver but low in human ESC and in kidney. (B) Hox A cluster 5hmC levels are low in HLC and human liver, but high in human ESC and kidney. Data were visualised by Integrated Genome Viewer (Broad Institute, <http://software.broadinstitute.org/software/igv/)>. The height of each histogram bar corresponds to the normalised 5hmC score per 150bp window at each locus.


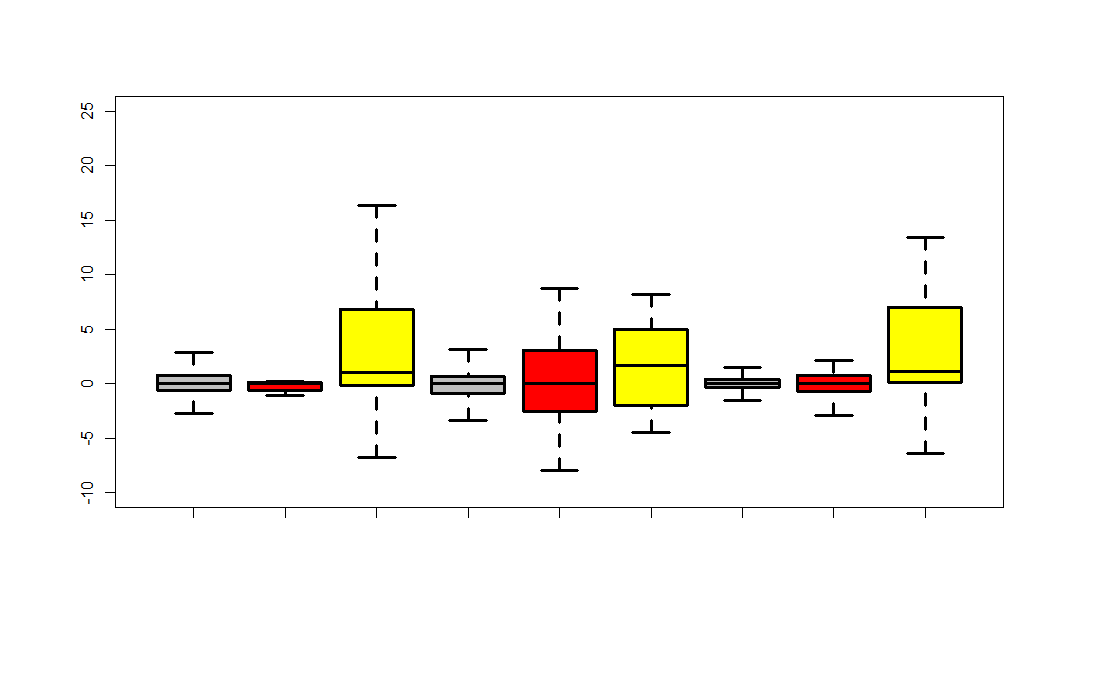


**All Genes**

**Other induced genes**

**Lipid synthesis and transport genes**

**All Genes**

**Other induced genes**

**Lipid synthesis and transport genes**

**All Genes**

**Other induced genes**

**Lipid synthesis and transport genes**

**TSS**

**Promoter**

**Gene Body**

*Supplementary Figure 6: Changes in mean genic 5hmC on LPO treatment*

Change in mean genic levels of 5hmC following LPO exposure in all genes (grey), induced genes mediating lipid synthesis and transport (yellow), other induced genes (red) in promoter, TSS and genic regions. Experiment is the mean of three separate hmeDIP sequencing experiments per treatment group.

**3. Supplementary tables**

*Supplemental Table 1: Primers*

| **RT qPCR Primer** | **Sequence** | Roche UPL probe |
| --- | --- | --- |
| ALB reverse | gtgaggttgctcatcggttt | 7 |
| ALB forward | gagcaaaggcaatcaacacc | 7 |
| POUF51 forward | caatttgccaagctcctga | 7 |
| POUF51 reverse | agatggtcgtttggctgaat | 7 |
| HNF4A forward | agcaacggacagatgtgtga | 27 |
| HNF4A reverse | tcagaccctgagccacct | 27 |
| NANOG forward | atgcctcacacggagactgt | 69 |
| NANOG reverse | cagggctgtcctgaataagc | 69 |
| PLIN1 forward | agagcgccagtagcttgg | 1 |
| PLIN1 reverse | ttggcagctgtgaactgg | 1 |
| PLIN2 forward | tcagctccattctactgttcacc | 72 |
| PLIN2 reverse | cctgaattttctgattggcact | 72 |
| PCK1 forward | agatggaggaagagggcatc | 41 |
| PCK1 reverse | ggtcagtgagagccaacca | 41 |
| PCK2 forward | cgaaagctccccaagtacaa | 20 |
| PCK2 reverse | gctctctactcgtgccacatc | 20 |
| G6PD forward | aacagagtgagcccttcttca | 5 |
| G6PD reverse | ggaggctgcatcatcgtact | 5 |
| FASN forward | catcggctccaccaagtc | 1 |
| FASN reverse | gctatggaagtgcaggttgg | 1 |
| LXR forward | gggcatgatcgagaagctc | 44 |
| LXR reverse | cggctatggggatctggt | 44 |
| ACACA forward | tcaaactgcaggtatcccaac | 1 |
| ACACA reverse | attttcctgccagtccacac | 1 |
| SREB1c forward | gtgggcactgaggcaaag | 2 |
| SREB1c reverse | gacagcagtgcgcagactta | 2 |

| **5hmC-DIP qPCR primer** | **Primer** |
| --- | --- |
| Gapdh Promoter forward | cggctactagcggttttacg |
| Gapdh Promoter reverse | aagaagatgcggctgactgt |
| Line-1 PA1 forward | aaatggtgctgggaaaactg |
| Line-1 PA1 reverse | gccattgcttttggtgtttt |
| UBIAD1 genic forward | ctcttcctcctcctcgtcct |
| UBIAD1 genic reverse | catccaggaaccacagtcct |

*Supplementary table 2 Microarray analysis of gene expression following 48 hour low dose LPO or control treatment of HLCs.* Genes with the greatest positive and negative derangement (1.5 fold change) reaching significance (p<0.05 BH correction) are shown.

| **Illumina probe_ID** | **symbol** | **logFC** | **adj.P.Val** |
| --- | --- | --- | --- |
| ILMN_2412336 | AKR1C2 | 2.18 | 0.0000 |
| ILMN_1687757 | AKR1C4 | 1.73 | 0.0000 |
| ILMN_1801077 | PLIN2 | 1.60 | 0.0000 |
| ILMN_1675706 | APOA4 | 1.50 | 0.0000 |
| ILMN_2174437 | CIDEC | 1.37 | 0.0000 |
| ILMN_2138765 | PLIN2 | 1.35 | 0.0000 |
| ILMN_2278335 | AKR1B15 | 1.15 | 0.0015 |
| ILMN_1672148 | AKR1B10 | 1.14 | 0.0004 |
| ILMN_2219681 | RBP2 | 1.10 | 0.0000 |
| ILMN_1815203 | HMGCS2 | 1.08 | 0.0000 |
| ILMN_2387385 | IGFBP1 | 1.03 | 0.0011 |
| ILMN_2124802 | MT1H | 1.02 | 0.0000 |
| ILMN_1684306 | S100A4 | 0.97 | 0.0491 |
| ILMN_2188862 | GDF15 | 0.95 | 0.0001 |
| ILMN_1750974 | S100A9 | 0.94 | 0.0223 |
| ILMN_2349393 | MDK | 0.93 | 0.0000 |
| ILMN_1754055 | APOA5 | 0.88 | 0.0002 |
| ILMN_1736178 | AEBP1 | 0.88 | 0.0000 |
| ILMN_1807291 | CYP1A1 | 0.85 | 0.0001 |
| ILMN_2108735 | EEF1A2 | 0.85 | 0.0001 |
| ILMN_1804822 | SRXN1 | 0.83 | 0.0004 |
| ILMN_1747067 | NPAS1 | 0.82 | 0.0000 |
| ILMN_1735816 | CYP4A11 | 0.81 | 0.0084 |
| ILMN_1713124 | AKR1C3 | 0.81 | 0.0000 |
| ILMN_1684982 | PDK4 | 0.79 | 0.0007 |
| ILMN_2212999 | KIF5C | 0.78 | 0.0001 |
| ILMN_1767470 | SCPEP1 | 0.77 | 0.0000 |
| ILMN_1666733 | CXCL8 | 0.77 | 0.0433 |
| ILMN_3307693 | WFDC2 | 0.77 | 0.0000 |
| ILMN_1715401 | MT1G | 0.76 | 0.0000 |
| ILMN_3249142 | ZG16 | 0.75 | 0.0045 |
| ILMN_2046073 | LCT | 0.74 | 0.0001 |
| ILMN_1684308 | DEFB103A | 0.73 | 0.0421 |
| ILMN_1717056 | TXNRD1 | 0.73 | 0.0000 |
| ILMN_2352009 | ACADVL | 0.73 | 0.0000 |
| ILMN_1829555 | LCE6A | 0.72 | 0.0092 |
| ILMN_1744817 | UGT1A1 | 0.71 | 0.0005 |
| ILMN_2121774 | ZG16 | 0.70 | 0.0049 |
| ILMN_1728445 | IGFBP1 | 0.69 | 0.0161 |
| ILMN_2388484 | MAP2 | 0.68 | 0.0001 |
| ILMN_1757387 | UCHL1 | 0.67 | 0.0000 |
| ILMN_1757406 | HIST1H1C | 0.66 | 0.0001 |
| ILMN_1704531 | PTGR1 | 0.66 | 0.0001 |
| ILMN_1758731 | CYP2J2 | 0.66 | 0.0000 |
| ILMN_1737298 | MAT2A | 0.65 | 0.0000 |
| ILMN_1789733 | CLIP3 | 0.64 | 0.0003 |
| ILMN_2324421 | TXNRD1 | 0.64 | 0.0006 |
| ILMN_1801216 | S100P | 0.64 | 0.0000 |
| ILMN_1779448 | EFHD1 | 0.64 | 0.0000 |
| ILMN_1731948 | PCK1 | 0.63 | 0.0069 |
| ILMN_2065773 | SCG5 | 0.63 | 0.0001 |
| ILMN_1707339 | BTG3 | 0.63 | 0.0002 |
| ILMN_1654262 | ZMAT3 | 0.62 | 0.0014 |
| ILMN_1653200 | SLC22A17 | 0.62 | 0.0001 |
| ILMN_1813175 | ADGRL1 | 0.62 | 0.0000 |
| ILMN_1794190 | CCPG1 | 0.61 | 0.0000 |
| ILMN_1700081 | FST | 0.61 | 0.0050 |
| ILMN_1786388 | RNF113A | 0.61 | 0.0012 |
| ILMN_1760414 | AADAC | 0.61 | 0.0005 |
| ILMN_1726682 | NA | 0.61 | 0.0000 |
| ILMN_1757467 | H1F0 | 0.60 | 0.0000 |
| ILMN_1657744 | FAM219B | 0.59 | 0.0000 |
| ILMN_2401344 | PPP2R2C | 0.59 | 0.0001 |
| ILMN_1763999 | NA | 0.58 | 0.0005 |
| ILMN_2398159 | DKK3 | 0.58 | 0.0000 |
| ILMN_1778144 | SLC48A1 | 0.58 | 0.0000 |
| ILMN_1744381 | SERPINE1 | 0.57 | 0.0035 |
| ILMN_1707727 | ANGPTL4 | 0.57 | 0.0002 |
| ILMN_1764201 | MAP2 | 0.57 | 0.0017 |
| ILMN_2305225 | NDRG4 | 0.56 | 0.0000 |
| ILMN_1679299 | IGSF1 | 0.56 | 0.0020 |
| ILMN_1651496 | HIST1H2BD | 0.56 | 0.0002 |
| ILMN_1728799 | FBP1 | 0.55 | 0.0002 |
| ILMN_1806403 | RASL12 | 0.55 | 0.0002 |
| ILMN_3246065 | CCDC151 | 0.55 | 0.0004 |
| ILMN_1758034 | ETFDH | 0.54 | 0.0001 |
| ILMN_2347592 | NMB | 0.54 | 0.0001 |
| ILMN_2095660 | TMEM156 | 0.54 | 0.0037 |
| ILMN_2374865 | ATF3 | 0.53 | 0.0020 |
| ILMN_1768721 | DPPA4 | 0.53 | 0.0002 |
| ILMN_3214389 | NA | 0.53 | 0.0000 |
| ILMN_1692661 | AKR1D1 | -0.53 | 0.0007 |
| ILMN_2380237 | C1QTNF1 | -0.53 | 0.0033 |
| ILMN_1808732 | SAA1 | -0.54 | 0.0004 |
| ILMN_1815205 | LYZ | -0.54 | 0.0179 |
| ILMN_1751814 | NA | -0.54 | 0.0003 |
| ILMN_3263974 | NA | -0.54 | 0.0026 |
| ILMN_1685079 | TELO2 | -0.54 | 0.0474 |
| ILMN_3226814 | NA | -0.54 | 0.0005 |
| ILMN_3191922 | NA | -0.55 | 0.0023 |
| ILMN_1686291 | DIO1 | -0.55 | 0.0003 |
| ILMN_1795190 | CLDN2 | -0.55 | 0.0010 |
| ILMN_1705261 | CAPN1 | -0.55 | 0.0002 |
| ILMN_1692861 | UGT2A3 | -0.55 | 0.0001 |
| ILMN_1780693 | HSD3B1 | -0.55 | 0.0064 |
| ILMN_1666638 | NA | -0.55 | 0.0001 |
| ILMN_1685115 | HEXIM1 | -0.55 | 0.0003 |
| ILMN_1727532 | OLFML3 | -0.55 | 0.0000 |
| ILMN_2218604 | KLHL41 | -0.55 | 0.0001 |
| ILMN_1662795 | CA2 | -0.55 | 0.0003 |
| ILMN_1912083 | NA | -0.56 | 0.0000 |
| ILMN_1810910 | CFH | -0.56 | 0.0099 |
| ILMN_2159730 | GABRB1 | -0.56 | 0.0003 |
| ILMN_1749829 | DLGAP5 | -0.56 | 0.0000 |
| ILMN_2169966 | TM4SF18 | -0.56 | 0.0108 |
| ILMN_1798992 | MYL3 | -0.56 | 0.0008 |
| ILMN_1737728 | CDCA3 | -0.57 | 0.0000 |
| ILMN_1770228 | KRT34 | -0.57 | 0.0016 |
| ILMN_2087656 | SLCO2B1 | -0.57 | 0.0017 |
| ILMN_1708110 | TMEM144 | -0.57 | 0.0000 |
| ILMN_1673566 | ADAMTS1 | -0.57 | 0.0001 |
| ILMN_1801939 | CCNB2 | -0.58 | 0.0001 |
| ILMN_1704247 | LAMA3 | -0.58 | 0.0004 |
| ILMN_1745491 | UNC5CL | -0.58 | 0.0026 |
| ILMN_2058251 | VIM | -0.58 | 0.0021 |
| ILMN_1670899 | FBN2 | -0.58 | 0.0004 |
| ILMN_1713807 | MAN1C1 | -0.58 | 0.0001 |
| ILMN_1698213 | RBM3 | -0.58 | 0.0000 |
| ILMN_1776905 | TMEM236 | -0.59 | 0.0000 |
| ILMN_1806387 | NA | -0.59 | 0.0001 |
| ILMN_1701613 | RARRES3 | -0.59 | 0.0001 |
| ILMN_3293685 | NA | -0.59 | 0.0004 |
| ILMN_1695658 | KIF20A | -0.60 | 0.0000 |
| ILMN_2179083 | LOXL4 | -0.60 | 0.0130 |
| ILMN_1728049 | S100A16 | -0.60 | 0.0000 |
| ILMN_1800573 | RPS21 | -0.60 | 0.0000 |
| ILMN_2218104 | PAH | -0.60 | 0.0002 |
| ILMN_1763837 | ANPEP | -0.61 | 0.0021 |
| ILMN_2213297 | C11orf54 | -0.61 | 0.0025 |
| ILMN_1687867 | NA | -0.61 | 0.0006 |
| ILMN_1734773 | PRSS1 | -0.61 | 0.0000 |
| ILMN_3252936 | NA | -0.62 | 0.0009 |
| ILMN_1813131 | NA | -0.62 | 0.0030 |
| ILMN_1683250 | NA | -0.62 | 0.0001 |
| ILMN_1753584 | KRT8 | -0.62 | 0.0001 |
| ILMN_1784364 | STARD5 | -0.62 | 0.0000 |
| ILMN_1671971 | NA | -0.62 | 0.0002 |
| ILMN_1728262 | SAA2 | -0.63 | 0.0006 |
| ILMN_1661595 | C1orf53 | -0.63 | 0.0000 |
| ILMN_1679194 | NA | -0.64 | 0.0039 |
| ILMN_3178307 | NA | -0.64 | 0.0005 |
| ILMN_2125869 | ACTA1 | -0.64 | 0.0000 |
| ILMN_1690866 | KLHL41 | -0.64 | 0.0001 |
| ILMN_1679262 | DPYSL3 | -0.64 | 0.0000 |
| ILMN_3251409 | PDE6A | -0.65 | 0.0000 |
| ILMN_1777325 | STAT1 | -0.65 | 0.0000 |
| ILMN_1702489 | TRIM63 | -0.65 | 0.0002 |
| ILMN_1760087 | SLC26A3 | -0.65 | 0.0025 |
| ILMN_1737517 | RPL29 | -0.65 | 0.0001 |
| ILMN_1791726 | TUBB3 | -0.65 | 0.0000 |
| ILMN_3280565 | NA | -0.65 | 0.0006 |
| ILMN_1788122 | GSTA5 | -0.66 | 0.0001 |
| ILMN_1716859 | TDO2 | -0.66 | 0.0007 |
| ILMN_1698246 | MTMR11 | -0.66 | 0.0020 |
| ILMN_1719543 | MAF | -0.67 | 0.0000 |
| ILMN_1754576 | KRT6C | -0.67 | 0.0186 |
| ILMN_1720710 | HSPB3 | -0.67 | 0.0004 |
| ILMN_2358074 | MAD1L1 | -0.67 | 0.0005 |
| ILMN_1755720 | SLC2A2 | -0.67 | 0.0019 |
| ILMN_2133205 | GPX2 | -0.67 | 0.0000 |
| ILMN_1696302 | FABP5 | -0.68 | 0.0000 |
| ILMN_2219002 | KRT6A | -0.68 | 0.0062 |
| ILMN_1789648 | SCGN | -0.69 | 0.0001 |
| ILMN_1707975 | SERPIND1 | -0.70 | 0.0015 |
| ILMN_3280842 | NA | -0.70 | 0.0003 |
| ILMN_1729251 | MYH4 | -0.70 | 0.0006 |
| ILMN_1661078 | NA | -0.71 | 0.0005 |
| ILMN_1755897 | NA | -0.71 | 0.0004 |
| ILMN_1779852 | NA | -0.72 | 0.0001 |
| ILMN_2389054 | CLDN18 | -0.73 | 0.0000 |
| ILMN_1671337 | SLC2A5 | -0.73 | 0.0001 |
| ILMN_1697922 | NA | -0.73 | 0.0001 |
| ILMN_1808677 | UGT2B17 | -0.73 | 0.0005 |
| ILMN_1771051 | RPL29 | -0.73 | 0.0001 |
| ILMN_1769547 | DIO1 | -0.74 | 0.0008 |
| ILMN_2414786 | DIO1 | -0.74 | 0.0008 |
| ILMN_1754247 | SLC3A1 | -0.75 | 0.0022 |
| ILMN_1662214 | PAH | -0.75 | 0.0003 |
| ILMN_3209399 | NA | -0.75 | 0.0000 |
| ILMN_1709847 | KCNJ13 | -0.75 | 0.0001 |
| ILMN_1691364 | STAT1 | -0.75 | 0.0000 |
| ILMN_1667018 | ACE2 | -0.75 | 0.0006 |
| ILMN_1905548 | MBNL3 | -0.76 | 0.0000 |
| ILMN_1803429 | CD44 | -0.76 | 0.0035 |
| ILMN_2400935 | TAGLN | -0.77 | 0.0003 |
| ILMN_1766675 | CDH6 | -0.77 | 0.0001 |
| ILMN_1694588 | C4BPB | -0.78 | 0.0000 |
| ILMN_1663390 | CDC20 | -0.79 | 0.0000 |
| ILMN_1706554 | NA | -0.79 | 0.0002 |
| ILMN_3178258 | NA | -0.79 | 0.0000 |
| ILMN_2207504 | LEP | -0.79 | 0.0014 |
| ILMN_2374449 | SPP1 | -0.82 | 0.0041 |
| ILMN_1690105 | STAT1 | -0.82 | 0.0000 |
| ILMN_1823750 | JAKMIP3 | -0.83 | 0.0002 |
| ILMN_1734176 | CGA | -0.83 | 0.0000 |
| ILMN_3266606 | NA | -0.83 | 0.0000 |
| ILMN_1778668 | TAGLN | -0.84 | 0.0001 |
| ILMN_2239408 | RNASE4 | -0.84 | 0.0000 |
| ILMN_1737041 | HABP2 | -0.84 | 0.0002 |
| ILMN_2146761 | FABP5 | -0.85 | 0.0000 |
| ILMN_3210741 | NA | -0.85 | 0.0000 |
| ILMN_1776112 | SLC10A1 | -0.86 | 0.0113 |
| ILMN_1716925 | FSIP1 | -0.86 | 0.0001 |
| ILMN_1651354 | SPP1 | -0.86 | 0.0073 |
| ILMN_2162819 | UGT2B11 | -0.87 | 0.0002 |
| ILMN_1670589 | NA | -0.87 | 0.0002 |
| ILMN_1872974 | NA | -0.88 | 0.0000 |
| ILMN_1704208 | NA | -0.88 | 0.0013 |
| ILMN_1771544 | FMO9P | -0.90 | 0.0092 |
| ILMN_1740443 | NA | -0.91 | 0.0004 |
| ILMN_1764690 | NTS | -0.92 | 0.0017 |
| ILMN_1808114 | LYVE1 | -0.92 | 0.0000 |
| ILMN_1810233 | UGT2B11 | -0.94 | 0.0008 |
| ILMN_3238960 | NA | -0.94 | 0.0000 |
| ILMN_3269324 | NA | -0.95 | 0.0000 |
| ILMN_1808494 | ITIH2 | -0.95 | 0.0041 |
| ILMN_1763359 | NA | -0.96 | 0.0000 |
| ILMN_1766955 | VCAM1 | -0.99 | 0.0000 |
| ILMN_1750234 | PRSS2 | -0.99 | 0.0000 |
| ILMN_1685699 | PRSS3 | -1.02 | 0.0000 |
| ILMN_2307903 | VCAM1 | -1.08 | 0.0001 |
| ILMN_1781859 | UGT2B28 | -1.09 | 0.0001 |
| ILMN_2297626 | PEG10 | -1.14 | 0.0000 |
| ILMN_1742444 | UGT2B10 | -1.20 | 0.0000 |
| ILMN_1699011 | NA | -1.23 | 0.0001 |
| ILMN_1777797 | AFM | -1.26 | 0.0000 |
| ILMN_1685043 | CYP3A7 | -1.31 | 0.0002 |
| ILMN_1678841 | UBD | -1.39 | 0.0000 |
| ILMN_1696284 | CLDN18 | -1.48 | 0.0000 |
| ILMN_1780575 | CRP | -1.80 | 0.0018 |

**4. Supplementary References**

1. Bray MA, Singh S, Han H, Davis CT, Borgeson B, Hartland C, Kost-Alimova M, et al. Cell Painting, a high-content image-based assay for morphological profiling using multiplexed fluorescent dyes. Nat Protoc 2016;11:1757-1774.

2. Payne CM, Samuel K, Pryde A, King J, Brownstein D, Schrader J, Medine CN, et al. Persistence of functional hepatocyte-like cells in immune-compromised mice. Liver Int 2011;31:254-262.

3. Smith CA, Want EJ, O'Maille G, Abagyan R, Siuzdak G. XCMS: processing mass spectrometry data for metabolite profiling using nonlinear peak alignment, matching, and identification. Anal Chem 2006;78:779-787.

4. Dunn WB, Broadhurst D, Brown M, Baker PN, Redman CW, Kenny LC, Kell DB. Metabolic profiling of serum using Ultra Performance Liquid Chromatography and the LTQ-Orbitrap mass spectrometry system. J Chromatogr B Analyt Technol Biomed Life Sci 2008;871:288-298.

5. Brown M, Wedge DC, Goodacre R, Kell DB, Baker PN, Kenny LC, Mamas MA, et al. Automated workflows for accurate mass-based putative metabolite identification in LC/MS-derived metabolomic datasets. Bioinformatics 2011;27:1108-1112.

6. Sumner LW, Amberg A, Barrett D, Beale MH, Beger R, Daykin CA, Fan TW, et al. Proposed minimum reporting standards for chemical analysis Chemical Analysis Working Group (CAWG) Metabolomics Standards Initiative (MSI). Metabolomics 2007;3:211-221.

7. Dunn WB, Broadhurst D, Begley P, Zelena E, Francis-McIntyre S, Anderson N, Brown M, et al. Procedures for large-scale metabolic profiling of serum and plasma using gas chromatography and liquid chromatography coupled to mass spectrometry. Nat Protoc 2011;6:1060-1083.

8. Xia J, Sinelnikov IV, Han B, Wishart DS. MetaboAnalyst 3.0--making metabolomics more meaningful. Nucleic Acids Res 2015;43:W251-257.

9. Li X, Liu Y, Salz T, Hansen KD, Feinberg A. Whole-genome analysis of the methylome and hydroxymethylome in normal and malignant lung and liver. Genome Res 2016;26:1730-1741.

10. Kim M, Park YK, Kang TW, Lee SH, Rhee YH, Park JL, Kim HJ, et al. Dynamic changes in DNA methylation and hydroxymethylation when hES cells undergo differentiation toward a neuronal lineage. Hum Mol Genet 2014;23:657-667.

11. Thomson JP, Fawkes A, Ottaviano R, Hunter JM, Shukla R, Mjoseng HK, Clark R, et al. DNA immunoprecipitation semiconductor sequencing (DIP-SC-seq) as a rapid method to generate genome wide epigenetic signatures. Sci. Rep. 2015;5.
